# LncRNA *DANCR* upregulates PI3K/AKT signaling through activating serine phosphorylation of RXRA

**DOI:** 10.1038/s41419-018-1220-7

**Published:** 2018-12-05

**Authors:** Jianming Tang, Guangsheng Zhong, Haibo Zhang, Bo Yu, Fangqiang Wei, Liming Luo, Yao kang, Jianhui Wu, Jiaxiang Jiang, Yucheng Li, Shuqiang Wu, Yongshi Jia, Xiaodong Liang, Aihong Bi

**Affiliations:** 1 Department of Radiation Oncology, Zhejiang Provincial People’ s Hospital, People’ s Hospital of Hangzhou Medical College, Hangzhou, Zhejiang 310014 P.R. China; 2 Department of Thyroid and Breast Surgery, Zhejiang Provincial People’ s Hospital, People’ s Hospital of Hangzhou Medical College, Hangzhou, Zhejiang 310014 P.R. China; 3 Department of Oncology, Taixing People’s Hospital, Taixing, Jiangsu 225300 P.R. China; 4 Department of Hepatobiliary and Pancreatic Surgery, Zhejiang Provincial People’ s Hospital, People’ s Hospital of Hangzhou Medical College, Hangzhou, Zhejiang 310014 P.R. China; 5 Department of Orthopaedic Surgery, Zhejiang Provincial People’ s Hospital, People’ s Hospital of Hangzhou Medical College, Hangzhou, Zhejiang 310014 P.R. China; 6 Department of The Otolaryngology, Meizhou People’s Hospital, Meizhou, Guangdong 514000 P.R. China; 7 Department of Cardiology, Zhejiang Provincial People’ s Hospital, People’ s Hospital of Hangzhou Medical College, Hangzhou, Zhejiang 310014 P.R. China

## Abstract

Conventional therapies and novel molecular targeted therapies against breast cancer have gained great advances over the past two decades. However, poor prognosis and low survival rate are far from expectation for improvement, particularly in patients with triple negative breast cancer (TNBC). Here, we found that lncRNA *DANCR* was significantly overregulated in TNBC tissues and cell lines compared with normal breast tissues or other type of breast cancer. Knockdown of *DANCR* suppressed TNBC proliferation both in vitro and in vivo. Further study of underlying mechanisms demonstrated that *DANCR* bound with RXRA and increased its serine 49/78 phosphorylation via GSK3β, resulting in activating PIK3CA transcription, and subsequently enhanced PI3K/AKT signaling and TNBC tumorigenesis. Taken together, Our findings identified *DANCR* as an pro-oncogene and uncoverd a new working pattern of lncRNA to mediate TNBC tumorigenesis, which may be a potential therapeutic target for improving treatment of TNBC.

## Introduction

Breast cancer is the most prevalent malignant tumor in women, and the efficacy of currently available therapies seems far from satisfactory, which severely threats the health of females from all over the world^1,2^. Generally, breast cancer can be classified into four subtypes, including luminal type A, luminal type B, human epidermal growth factor receptor-2 (HER2) positive type, and triple-negative breast cancer (TNBC) type^3^. TNBC demonstrates a lack of ER, PR, and HER2 expression by immunohistochemical results, and acts as a highly invasive subtype comprising about 20% of all breast cancer patients^4,5^. Furthermore, TNBC can exhibit high invasiveness, metastasis, high recurrence risk, and mortality rates, resulting in poor prognosis^6^. The lack of molecular targeted therapies and the poor survival of TNBC patients have fostered great endeavors to discover precise molecular targets for clinical treatment strategies. A growing body of evidence suggests that long non-coding RNAs (LncRNAs) are involved in TNBC progression through regulating tumor-related gene expression^7–9^. However, the precise molecular mechanisms by which lncRNA mediates TNBC progression remain unclear.

Differentiation antagonizing non-protein coding RNA (*DANCR*), an lncRNA encoded on human chromosome 4q12, has been identified as an oncogene in multiple malignant tumors, including colon cancer^10^, esophageal cancer^11^, hepatocellular carcinoma^12^, osteosarcoma^13^, TNBC^14^. DANCR was previously demonstrated to contribute to suppression of cell differentiation due to acting as a negative regulator^15,16^. Recent studies demonstrated that *DANCR* directly interacts with miR-758-3p^17^ and miR-577^18^ in non-small cell lung cancer and colorectal cancer, respectively. *DANCR* represses the expression of TIMP2/3 through physical binding with EZH2 in prostate cancer^19^. *DANCR* is also shown as a direct target of MYC in cancer^20^. Furthermore, *DANCR* expression is correlated with survival and/or prognosis of patients with hepatocellular carcinoma^12^, gastric cancer^21^, and colorectal cancer^10^. However, due to the molecular and phenotypic heterogeneity within and between different tumor types, mechanism explorations are required to elucidate the precise biological behaviour of *DANCR* in tumors, especially TNBC.

Here, we showed that *DANCR* is more highly expressed in TNBC compared with that in normal breast tissues, which exhibits poor prognosis. *DANCR* promoted proliferation and tumorigenesis in TNBC through activating ser49/78 phosphorylation of RXRA, and thus promoting PIK3CA expression. We also showed that signaling axis *DANCR-*RXRA-PI3K/AKT plays important roles in TNBC proliferation and tumorigenesis in vitro and vivo, respectively. In conclusion, the present study elucidated the function of *DANCR* in TNBC and might provide a novel of signaling pathway in the treatment of TNBC.

## Materials and methods

### Clinical samples

Between August 2013 and August 2015, clinical specimens containing breast cancer tissues including 60 triple-negative (TNBC) type, 15 HER2 type, 15 Luminal A type, and 15 Luminal B type, and 10 normal breast tissues were obtained from department of Breast Surgery in Meizhou People’ s Hospital. All these patients had not received chemotherapy and radiotherapy before the operation and all clinical samples were confirmed by pathology. This study protocol approval from the Research Ethics Committee of the Meizhou People’ s Hospital, and written informed consent from each participant were obtained.

### Cell lines

BT549, MCF7, T47D, MDA-MB-231, MDA-MB-453, and MDA-MB-468 cells were purchased from Cell Bank of the Chinese Scientific Academy (Shanghai, China), and were cultured in Dulbecco’ s modified Eagle’ s medium (DMEM) (Hyclone, Life Technologies, CA) with 10% fetal bovine serum (Hyclone, Life Technologies, CA). MCF10A was also from Cell Bank of the Chinese Scientific Academy (Shanghai, China), and was cultured in DMEM/F12 (Hyclone, Life Technologies, CA) supplemented with 5% horse serum (Invitrogen, Carlsbad, CA), 20 ng/ml hEGF (Sigma-Aldrich), 0.5 μg/ml hydrocortisone (Sigma-Aldrich), 100 ng/ml cholera toxin (Sigma-Aldrich),10 μg/ml insulin (Sigma-Aldrich), and 100 U/ml penicillin-streptomycin (Sigma-Aldrich). BT549, MCF7, T47D, MDA-MB-231, MDA-MB-453, MDA-MB-468, and MCF10A were recently authenticated through using STR DNA fingerprinting from Shanghai Biowing Applied Biotechnology Co., Ltd. Moreover, by using LookOut Mycoplasma PCR Detection kit (Sigma-Aldrich), we detected the mycoplasma infection.

### RNA extraction and quantitative RT-PCR

Total RNA was isolated using TRIzol reagent (Invitrogen, Carlsbad, CA, USA) and extracted according to the manufacturers’ protocol (Sigma-Aldrich). Reverse transcription (RT) was performed using the M-MLV Reverse Transcription Kit(Thermo Fisher Scientific). Quantitative RT–PCR was performed by means of Power qPCR Premix (SYBR Green) (Shang hai Generay Biotech CoCo., Ltd). GAPDH was used as a control. Primers are contained in Supplenentary Table S1.

### Western blot analysis

Western blot was performed as we previously described^22^. The specific antibodies for WB as follows: anti-RXRA (21218-1-AP, 1:1000, Proteintech), Phospho-RXRA (PA5-64630,1:1000, Invitrogen), GSK3β (ab65740, 1:1000, Abcam), Akt (#4685S, 1:1000, Cell Signaling Technology), Phospho-Akt (#4060S, 1:1000, Cell Signaling Technology), PIK3CA (MA5-17149,1:1000, Thermo Fisher), GAPDH (#5174S, 1:1000, Cell Signaling Technology).

### ChIP-qPCR

ChIP assay was performed using EZ-Magna ChIP™ A/G Chromatin Immunoprecipitation Kit (Millipore-17-408) referring to the manufacturer’s protocol.

Input genomic DNA and the purified immunoprecipitated DNA were used for qRT-PCR. Primers are contained in Supplementary Table 1.

### Luciferase promoter assay

pGL3-PIK3CA promoter wild or mutant type of the RXRA binding was performed co-transfection with or without RXRA using Lipofectamine RNAiMAX Reagent (Thero Fisher) referring to the manufacturer’s protocol. pRL Renilla luciferase control reporter vector (Promega) was presented as a control. A dual-luciferase assay was analyzed 48 h after co-transfection using the Promega E1960 Dual-Luciferase® Reporter System in accordance with the manufacturer recommendation.

### RNA immunoprecipitation (RIP) and RNA pull-down assays

The EZ-Magna RIP Kit (17-701, Millipore) was used for RIP assay with 10 μg anti-RXRA (21218-1-AP, Proteintech) referring to the manufacturer recommendation. The purified immunoprecipitated RNA and input genomic RNA were detected by quantitative RT-PCR. Biotin-labeled RNA was transcribed with Biotin RNA Labeling Mix (Roche 11685597910) and T7 RNA polymerase (Roche 10881775001), mixed with DNase I recombinant (Roche 04716728001), and purified with RNeasy Mini Kit (Qiagen 74904). Cell nuclear proteins were extracted using the pierce 78833 NE-PER (R) Nuclear and Cytoplasmic Extraction Reagents. Cell nuclear extract was mixed with Biotin-labeled RNA. Washed streptavidin agarose beads (Sigma-Aldrich) were added to each reaction. The binding protein was analyzed by Western blot assay.

### Cell proliferation and soft agar colony formation

A WST-1 Assay Kit (Roche) was used for cell proliferation assay. Cells were seeded in a 48-well plate and then incubated at 37 °C. Cell numbers were assessed with the WST-1 Assay Kit. For soft agar colony formation, cells were were split into the suspension of a single-cell, and then seeded in a media containing 0.4% top layer agar and 0.8% bottom layer agar in a 6-well plate. Cell culture media was changed every 4 days after seeding. Colonies were fixed with 4% paraformaldehyde and stained with 2% crystal violet solution after 2–3 weeks, respectively. The visible colony numbers scored and data were analyzed.

### Construction of vectors

The cDNA encoding *DANCR*, PIK3CA,GSK3β, RXRA were amplified from MCF10A cells and sequenced, and then subcloned into the pcDNA3 vector (Invitrogen), subsequently named pCDNA3-DANCR, pCDNA3-PIK3CA, pCDNA3-GSK3β, pCDNA3-RXRA. pLVX-DANCR, pLVX-PIK3CA, pLVX-GSK3β, and pLVX-RXRA was generated from pCDNA3-DANCR, pCDNA3-PIK3CA, pCDNA3-GSK3β, pCDNA3- RXRA, respectively. PIK3CA promoter was PCR-amplified from MCF10A cells and sequenced, and then subcloned into pGL3 vector (Promega). A Quik Change Site-Directed Mutagenesis Kit (Stratagene) was used for point mutations. shRNAs for *DANCR* were designed (shDANCR-1 target sequence: 5′-GGAGCTAG AGCAGTGACAATG-3′; shDANCR-2 target sequence 5′-GGTCACCAGACTTGCT ACACC-3′), and RXRA (shRXRA target sequence: 5′-GGCAAGCACTATGG AGTGTAC-3′, respectively.

### Tumorigenesis studies

BALB/c-nude mice female at an age of 4–5 weeks (SLAC, Shanghai, China) were randomly divided into 4 per group, and then MDA-MB-231 cells (3 × 10^6^) were implanted subcutaneously into mammary fat pads of each mice. All animal experiments procedures were approved by Zhejiang Provincial People’s Hospital the Guidance of Institutional Animal Care and Use Committee (IACUC). The IVIS Lumina imaging station (Caliper Life Sciences) was used for bioluminescence imaging. Different investigators independently performed mice allocation, surgery and the outcome assessing.

### Statistical analysis

All statistical analyses were performed using the GraphPad Prism version 5.0. The significance of the data from patient specimens was determined by Pearson’ s correlation coefficient. The significance of data from the vitro and vivo between experimental groups was determined by the Student’ s test or Mann–Whitney *U*-test *P < 0.05 was considered statistically significant.

## Results

### Expression of *DANCR* in clinical TNBC specimens

**Fig. 1:**
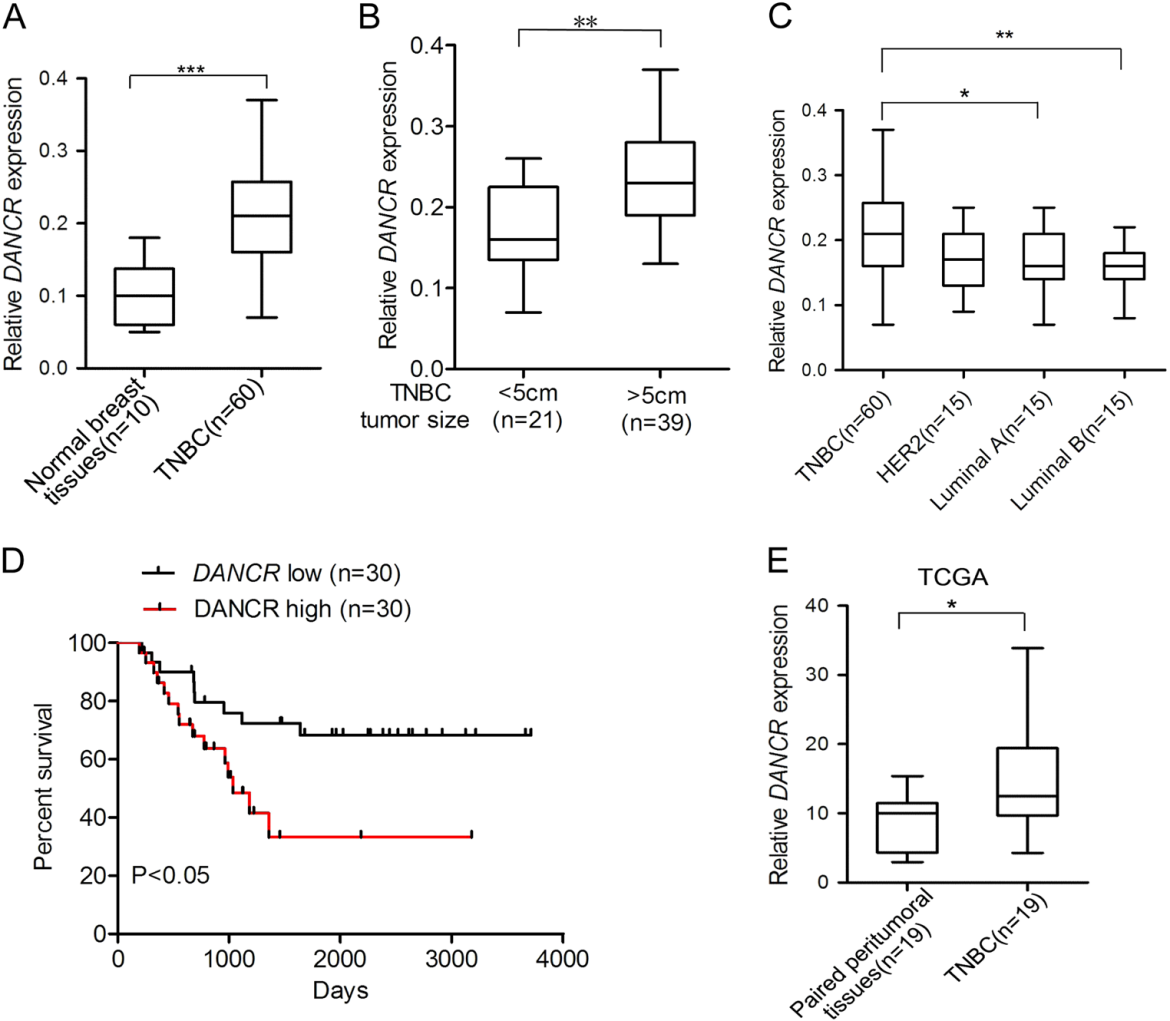
Expression of *DANCR* in clinical TNBC specimens. **a** Expression levels of *DANCR* mRNA are markedly higher in TNBC samples compared with normal breast tissues. **b** *DANCR* mRNA expression in clinical TNBC specimens with different tumor size groups (>5 cm and <5 cm). **c** Expression level of *DANCR* mRNA in breast cancers with TN (triple negative), Her2, Luminal A, and Luminal B types. **d** Kaplan–Meier analysis of patients with high *DANCR* mRNA-expressing TNBC versus low *DANCR* mRNA -expressing TNBC. Statistical analysis was performed by log-rank test in a GraphPad Prism version 5.0 for Windows. **e** *DAN*CR mRNA expression levels are markedly higher in clinical TNBC samples as compared to paired peritumoral breast tissues. Data of DANCR mRNA expression were downloaded from the Cancer Genome Atlas (TCGA) RNA-seq dataset. Error bars ± SD. *P < 0.05. **P < 0.01. ***P < 0.001. Data are representative from two independent experiments

We examined *DANCR* expression using clinical samples consisting of TNBC tissues and normal breast tissues. *DANCR* was found to be significantly upregulated in TNBC tumor tissues compared with that in normal breast tissues (Fig. 1a). To support our finding, we downloaded the TCGA RNA-seq dataset and microarray dataset of invasive breast cancer specimens, respectively. In these datasets, There was a clear trend that the TNBC tissues exhibits higher *DANCR* expression compared to the paired peritumoral tissues (Fig. 1e and Supplementary Figure 1A). Interestingly, higher level of *DANCR* was demonstrated to be associated with bigger tumor size (Fig. 1b). We then detected *DANCR* expression in breast cancer samples of various subtypes. *DANCR* showed significantly higher expression level in TNBC than that in the other subtypes of breast cancer (Fig. 1c). Furthermore, patients with high *DANCR* expression (*n* = 30) suffered poorer overall survival (OS) as compared to low expression group (*n* = 30) (Fig. 1d). Similarly, TCGA microarray dataset also demonstrated the result (Supplementary Figure 1B). Gene amplifications in DANCR were observed in mRNA overexpression (TCGA database from cBioPortal) (Supplementary Figure 1C). Taken together, this data demonstrated that *DANCR* is amplified and overexpressed in TNBC tumors.

### *DANCR* inhibition suppressed cell proliferation and tumor growth in TNBC

**Fig. 2:**
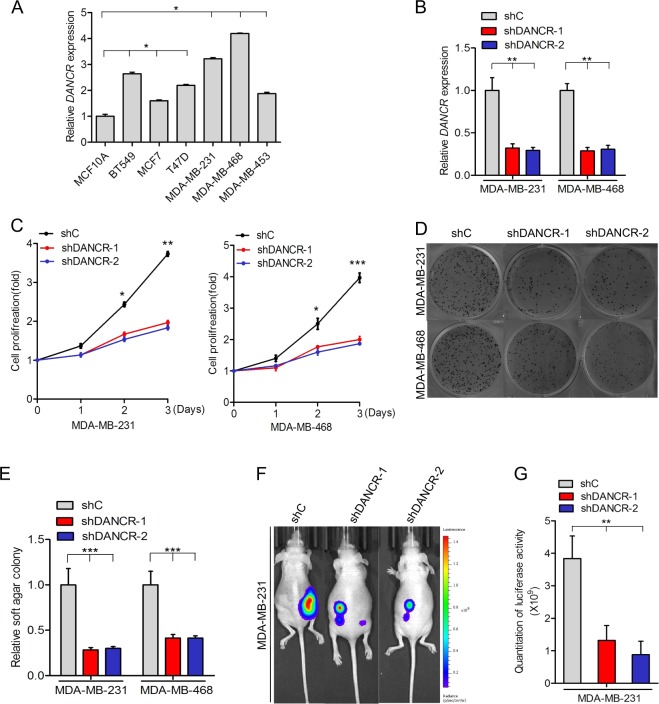
*DANCR* inhibition suppressed cell proliferation and tumor growth in TNBC. **a** qRT-PCR analysis of *DANCR* mRNA levels in breast normal and cancer cells. **b** qRT-PCR analysis of *DANCR* mRNA levels knockdown using two different shRNAs (shDANCR-1 and shDANCR-2) or a control shRNA (shC) in both MDA-MB-231 and MDA-MB-468 cells. **c**, **d** Effects of *DANCR* knockdown on TNBC cell proliferation (**c**) and soft agar colony formation (**d**). **e** Quantification of soft agar colony formation in D. **f** Representative bioluminescence images of shDANCR or shC-transfected MDA-MB-231 cells injected into the mice mammary gland fat pads. Mice were imaged at 4 weeks after transplantation. Data were from two independent experiments with 4 mice per group with similar results. **g** Quantification of bioluminescence activity in (**f**). Error bars ± SD. *P < 0.05. **P < 0.01. ***P < 0.001. Data are representative from two independent experiments

To further demonstrate the involvement of *DANCR* in TNBC, we tested *DANCR* expression in six breast cancer cell lines (BT549, MCF7, T47D, MDA-MB-231, MDA-MB-453, and MDA-MB-468) and a normal mammary epithelial cell line (MCF10A). *DANCR* expression was markedly upregulated in breast cancer cell lines as compared to that in MCF10A cell lines (Fig. 2a). Importantly, among all the six cancer cell lines, *DANCR* exhibited highest expression levels in two TNBC cell lines (MDA-MB-231 and MDA-MB-468).

We then tested the effect of *DANCR* inhibition on TNBC cell growth. Here, we knocked down the DANCR expression through treating MDA-MB-231 and MDA-MB-468 cells with the short hairpin RNA-mediated *DANCR* silencing (shRNA) or non-silencing control (shC), and found that depletion of endogenous *DANCR* significantly suppressed cell proliferation in both TNBC cell lines compared with the controls (Fig. 2b, c). Moreover, *DANCR* knockdown also impaired soft agar colony formation in MDA-MB-231 and MDA-MB-468 cells (Fig. 2d, e).

To further elucidate the TNBC tumorigenesis with *DANCR*, analysis of the orthotopic breast cancer model was performed. MDA-MB-231 cell lines transduced with shDANCR-1, shDANCR-2 or shControl (shC) were separately injected into the mice mammary gland fat pads. Remarkably, knockdown of *DANCR* was significantly decreased TNBC tumor growth compared with controls (Fig. 2f, g). These data suggested that *DANCR* contributes to TNBC cell proliferation and tumor growth.

### *DANCR* interacts with RXRA in TNBC cells

**Fig. 3:**
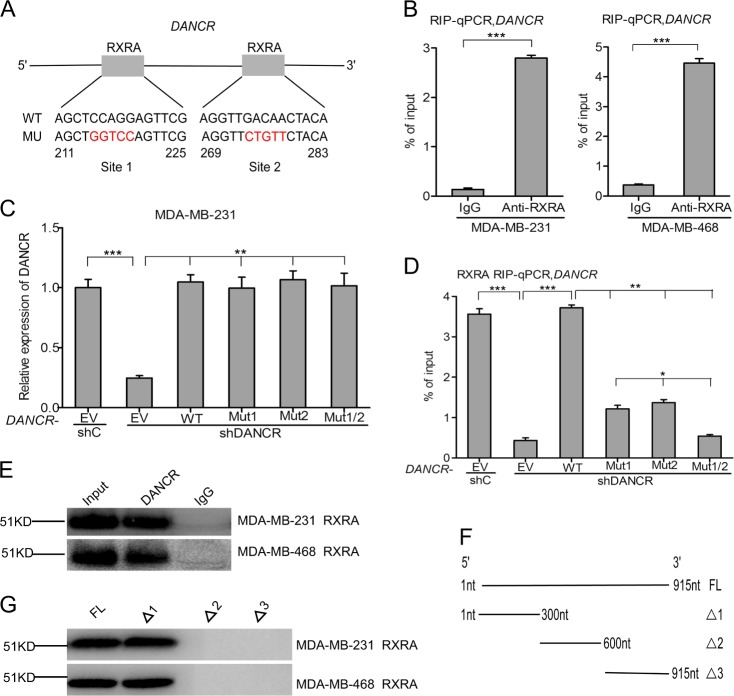
*DANCR* interacts with RXRA in TNBC cells. **a** Schematic diagram of putative RXRA binding sites in *DANCR*. **b** RIP-qPCR assay of the association of RXRA with *DANCR* in MDA-MB-231 and MDA-MB-468 cells. **c** Re-expression of shRNA-resistant *DANCR* wild type and RXRA-binding mutant types. **d** RIP-qPCR assay of effects of re-expression of shRNA-resistant *DANCR* wild type or mutant types on RXRA binding. **e** Biotinylated *DANCR* was incubated with nuclear extracts (MDA-MB-231 and MDA-MB-468 cells), targeted with streptavidin beads, and binding proteins were resolved in a gel. Western blotting assay of the specific binding of RXRA and *DANCR*. **f**, **g** RNAs corresponding to fragments in different regions of *DANCR* were treated as in (**e**), and binding RXRA was detected by western blotting assay. Error bars ± SD. *P < 0.05. **P < 0.01. ***P < 0.001. Data are representative from two independent experiments

Recent studies have demonstrated that lncRNAs mainly function as sponges to bind functional proteins and then influence their downstream genes expression^23–25^. Thus, We hypothesized that *DANCR*-regulated tumorigenesis depends on its binding proteins. *DANCR* was examined for transcription factor binding sites using a JASPAR database of transcription factor binding profiles, which identified two RXRA-binding sites as the most potential candidates. It is predicted that RXRA may bind with *DANCR* at both 211 to 225 and 269 to 283 sites (Fig. 3a). To test this, we performed RIP quantitative PCR with an antibody against RXRA from nuclear extracts of both MDA-MB-231 and MDA-MB-468 cells. We demonstrated that *DANCR* specifically bound to endogenous RXRA protein (Fig. 3b).

We next constructed *DANCR* plasmids containing a wild type and three mutant types including *DANCR*-RXRA binding site1 (Mut1), site2 (Mut2), or both sites (Mut1/2). As we predicted, re-expression of shRNA-resistant *DANCR* cDNA encoding the wild type restored the binding of *DANCR* with RXRA, whereas re-expression of a *DANCR* shRNA-resistant plasmid containing the *DANCR*-RXRA binding site1, site 2, or two sites mutants did not rescue it, suggesting that these two sites are critical for *DANCR*-RXRA binding (Fig. 3c, d).

To further validate the binding between *DANCR* and RXRA, we performed RNA pulldown (Fig. 3e), and deletion-mapping methods (Fig. 3f, g) to demonstrate whether RXRA would bind within specific regions of *DANCR*. These data identified a 300nt region at the 5′ end of *DANCR* required for the binding with RXRA (Fig. 3g). Taken together, the RIP, RNA pulldown, and deletion-mapping data validate a specific binding between RXRA and *DANCR*.

### *DANCR* regulates RXRA phosphorylation

**Fig. 4:**
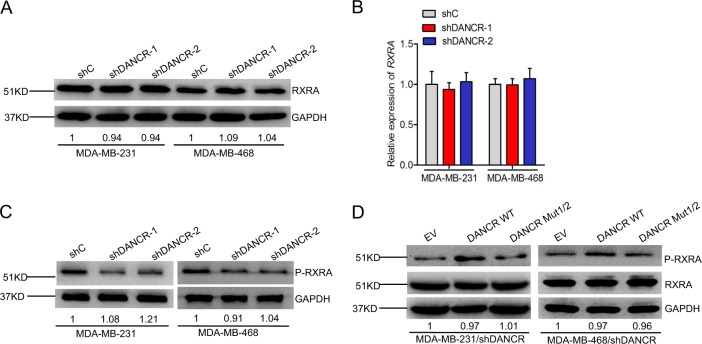
*DANCR* regulates RXRA phosphorylation. **a**, **b** WB analysis of effects of *DANCR* knockdown on RXRA protein (A) and mRNA (B) expression. **c** Effects of *DANCR* depletion on RXRA phosphorylation. **d** Effects of re-expression of shRNA resistant *DANCR* wild type or mutant type of the RXRA binding on RXRA phosphorylation

To explore the role of RXRA in *DANCR*-mediated TNBC tumor growth, we first assessed RXRA protein and expression in *DANCR* depletion TNBC cells. We found that *DANCR* knockdown did not affect the protein level of RXRA in MDA-MB-231 and MDA-MB-468 cells (Fig. 4a). Moreover, *DANCR* depletion had no effect on RXRA mRNA level (Fig. 4b). However, Western blot analysis showed that lower phosphorylation level of RXRA was found in the *DANCR*-knockdown group as compared to that of the controls (Fig. 4c). Furthermore, re-expression of shRNA resistant *DANCR* wild type rescued *DANCR* knockdown-inhibited RXRA phosphorylation, whereas re-expression of shRNA resistant *DANCR* mutant type of the RXRA binding did not affect it (Fig. 4d). Together, Our data suggest that *DANCR* enhance RXRA protein phosphorylation in TNBC.

### *DANCR* facilitates PIK3CA transcription in a RXRA-mediated manner

**Fig. 5:**
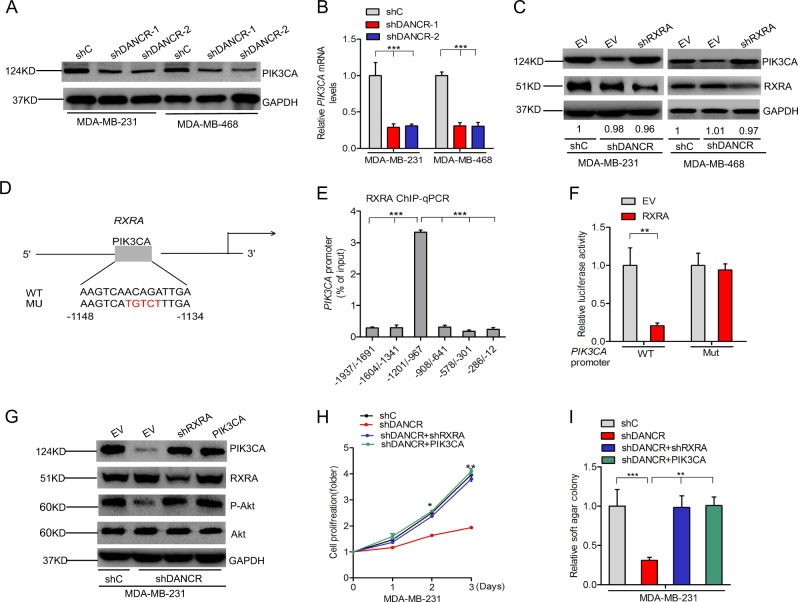
*DANCR* facilitates PIK3CA transcription in a RXRA-mediated manner. **a**, **b** Effects of *DANCR* knockdown on PIK3CA protein (**a**) and mRNA (**b**) expression in MDA-MB-231 and MDA-MB-468 cells. **c** Knockdown of RXRA restores *DANCR* depletion-inhibited PIK3CA expression in MDA-MB-231 and MDA-MB-468 cells. **d** Schematic diagram of the putative RXRA binding site in PIK3CA promoter. **e** ChIP-qPCR assay of the binding of RXRA with the PIK3CA promoter in MDA-MB-231 cells. **f** Luciferase assay of RXRA mediation on the PIK3CA promoter activity in MDA-MB-231 cells. **g** Knockdown of RXRA and overexpression of PIK3CA in *DANCR* depletion cells in MDA-MB-231 cells. **h**, **i** Knockdown of RXRA and overexpression of PIK3CA rescue *DANCR* knockdown-inhibited cell proliferation (**h**) and soft agar colony formation (**i**) in MDA-MB-231 cells. Error bars ± SD. *P < 0.05. **P < 0.01. ***P < 0.001. Data are representative from two independent experiments

Since PI3K signaling is critical for TNBC proliferation and Akt is a dominant downstream effector of PI3K signaling pathway^26^, we explored the effects of *DANCR* depletion on expression of phosphatidylinositol-4,5-biophosphate 3-kinase catalytic subunit alpha (PIK3CA) in MDA-MB-231 and MDA-MB-468 cells. As shown in Fig. 5a, b, knockdown of *DANCR* significantly inhibited PIK3CA protein and mRNA expression levels in both TNBC cells. However, knockdown of RXRA significantly rescued *DANCR* depletion-inhibited PIK3CA expression (Fig. 5c). These data suggest that *DANCR* may mediates RXRA to regulate PIK3CA expression.

To validate that RXRA regulates PIK3CA expression in TNBC cells, we predicted RXRA-binding sites to the promoter of PIK3CA using the JASPAR database of transcription factor binding profiles. One RXRA binding site was detected in the promoter of PIK3CA at the −1148 to −1134 site (Fig. 5d). RXRA Knockdown increased PIK3CA mRNA levels by qRT-PCR assays (Supplementary Figure 3). ChIP-qPCR assays using the antibody against RXRA demonstrated that RXRA bind to PIK3CA promoter (Fig. 5e). Promoter luciferase assays further validated that RXRA overexpression markedly decreased PIK3CA promoter transcriptional activity compared with the control, whereas the transcriptional activity was definitely restored after the mutation of RXRA binding site in PIK3CA promoter(Fig. 5f). Taken together, these data indicate that RXRA acts as a transcription repressors to inhibit PIK3CA expression.

To validate that *DANCR/*RXRA/PIK3CA signaling pathway regulates TNBC tumor growth, we down-regulated RXRA and overexpressed PIK3CA in MDA-MB-231 and MDA-MB-468 cells with a RXRA shRNA or a PIK3CA vector (Fig. 5g and Supplementary Figure 2A). Knockdown of RXRA rescued *DANCR* depletion-inhibited PIK3CA and Akt phosphorylation (Fig. 5g and Supplementary Figure 2A), cell proliferation (Fig. 5h and Supplementary Figure 2B), colony formation (Fig. 5i and Supplementary Figure 2C). Moreover, overexpression of PIK3CA restored Akt phosphorylation (Fig. 5g and Supplementary Figure 2A), cell proliferation (Fig. 5h and Supplementary Figure 2B), colony formation (Fig. 5i and Supplementary Figure 2C) inhibited by *DANCR* knockdown. Furthermore, *DANCR* expression was found to be correlated with PIK3CA expression (Supplementary Figure 2D). These data further demonstrate that *DANCR* mediates RXRA to upregulate PIK3CA expression, resulting in enhancing PI3K/AKT signaling pathway and promoting TNBC tumor growth.

### *DANCR*-mediated RXRA phosphorylation depends on GSK3β

**Fig. 6:**
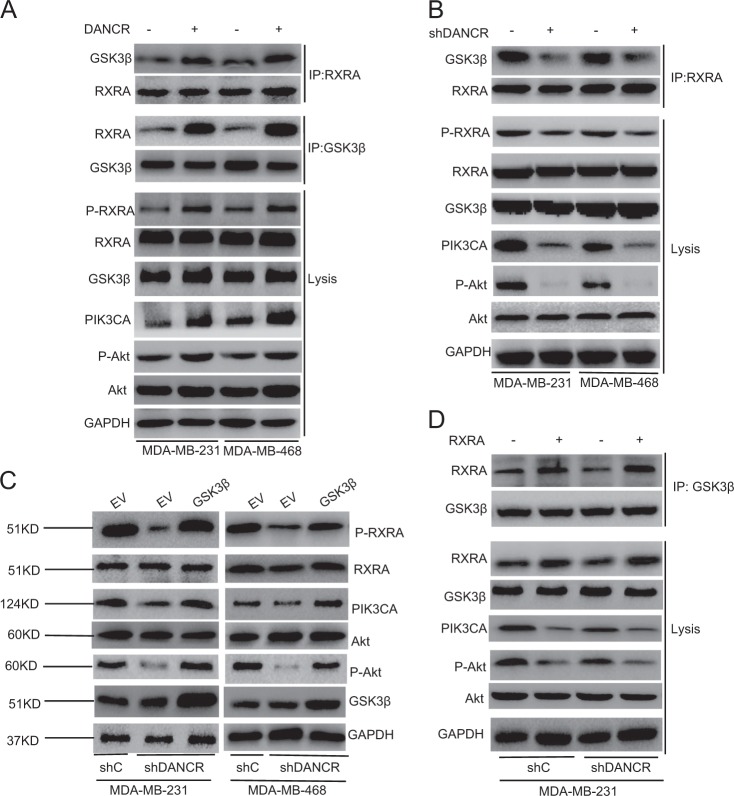
*DANCR*-mediated RXRA phosphorylation depends on GSK3β. **a** Effects of *DANCR* overexpression on RXRA-GSK3β binding and RXRA phosphorylation expression in MDA-MB-231 and MDA-MB-468 cells. **b** Effects of *DANCR* depletion on RXRA-GSK3β binding and RXRA phosphorylation expression. **c** GSK3β overexpression restored *DANCR* knockdown-inhibited RXRA phosphorylation. **d** Effects of overexpression of RXRA on RXRA-GSK3β binding in MDA-MB-231/shC and MDA-MB-231/shDANCR cells

Since the glycogen synthase kinase 3 beta (GSK3β) had been reported to promote RXRA phosphorylation in colorectal cancer cells^27^, we detected whether *DANCR*-regulated RXRA phosphorylation depends on GSK3β. As shown in Fig. 6a, compared with the control group, overexpression of *DANCR* increased RXRA binding with GSK3β, RXRA phosphorylation, Akt phosphorylation and PIK3CA expression in both TNBC cells (Fig. 6a). Knockdown of *DANCR* decreased RXRA binding with GSK3β, RXRA phosphorylation, Akt phosphorylation and PIK3CA expression in both TNBC cells (Fig. 6b). Furthermore, overexpression of GSK3β increased RXRA phosphorylation, Akt phosphorylation and PIK3CA expression (Fig. 6c) inhibited by *DANCR* knockdown. Consistent with previous study^27^, overexpression of RXRA increased its binding with GSK3β and decreased Akt phosphorylation and PIK3CA expression in MDA-MB-231 and MDA-MB-468 cells (Fig. 6d). Overexpression of RXRA significantly restored GSK3β association and Akt phosphorylation and PIK3CA expression inhibited by *DANCR* knockdown (Fig. 6d). These data support that *DANCR* depends on GSK3β to mediate RXRA phosphorylation in both TNBC cells.

### Ser49/Ser78 sites of RXRA protein are critical for DANCR-mediated TNBC tumor growth

**Fig. 7:**
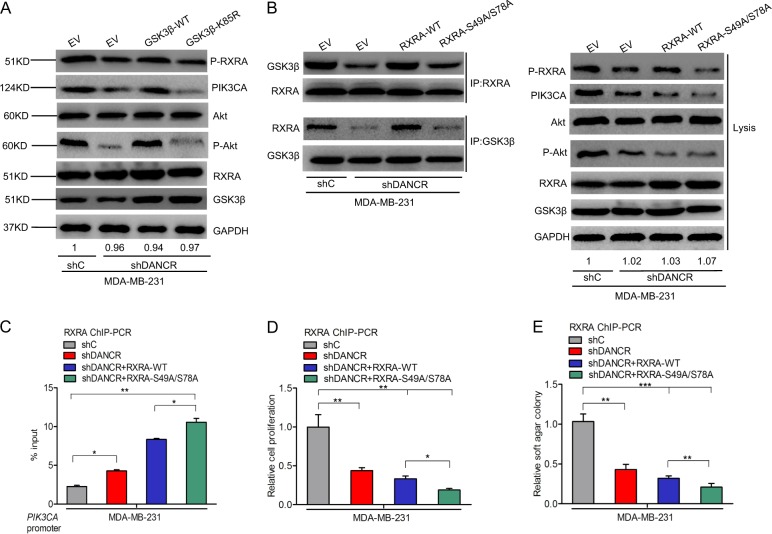
Ser49/Ser78 sites of RXRA protein are critical for DANCR-mediated TNBC tumor growth. **a** Effects of overexpression of GSK3β wild type or kinase deficient mutant type on RXRA phosphorylation and PI3K/Akt signaling activity in MDA-MB-231/shC and MDA-MB-231/shDANCR cells. **b** Effects of overexpression of RXRA wild type or mutant type on RXRA-GSK3β binding on RXRA phosphorylation and PI3K/Akt signaling activity in MDA-MB-231 with shC and shDANCR cells. **c**–**e** Effects of overexpression of RXRA wild type or mutant type on RXRA-GSK3β binding on RXRA protein binding PIK3CA ability (**c**), cell proliferation (**d**), and soft agar colony formation (**e**). Error bars ± SD. *P < 0.05. **P < 0.01. ***P < 0.001. Data are representative from two independent experiments

Since the roles of RXRA phosphorylation mediated by *DANCR* in TNBC is not well understood, we focused on the role of RXRA phosphorylation in *DANCR*-regulated TNBC cell proliferation. To detect whether RXRA phosphorylation is necessary to activate PI3K/AKT signaling pathway, we generated the GSK3β kinase deficient mutant plasmid, GSK3β-K58A. As shown Fig. 7a, overexpression of GSK3β cDNA encoding the wild type restored *DANCR* knockdown-inhibited RXRA phosphorylation, Akt phosphorylation and PIK3CA expression, whereas transfection of GSK3β kinase deficient mutant type did not rescue it. Next, we predicted potential serine phosphorylation sites of the RXRA protein using the Phosphor Motif Finder Program. Two serine phosphorylation candidate sites at Ser49 and Ser78 were identified. Transfection of RXRA wild type in MDA-MB-231 cells restored its binding with GSK3β inhibited by *DANCR* knockdown, and further decreased *DANCR* depletion-inhibited Akt phosphorylation, PIK3CA expression, cell proliferation, and soft agar colony formation (Fig. 7b–e). However, transfection of RXRA^S49A/S78A^ mutant did not rescued it compared with the wild type group (Fig. 7b–e). Taken together, these data demonstrates that serine phosphorylation of RXRA plays a crucial role in activating the PI3K/AKT signaling pathway.

## Discussion

**Fig. 8:**
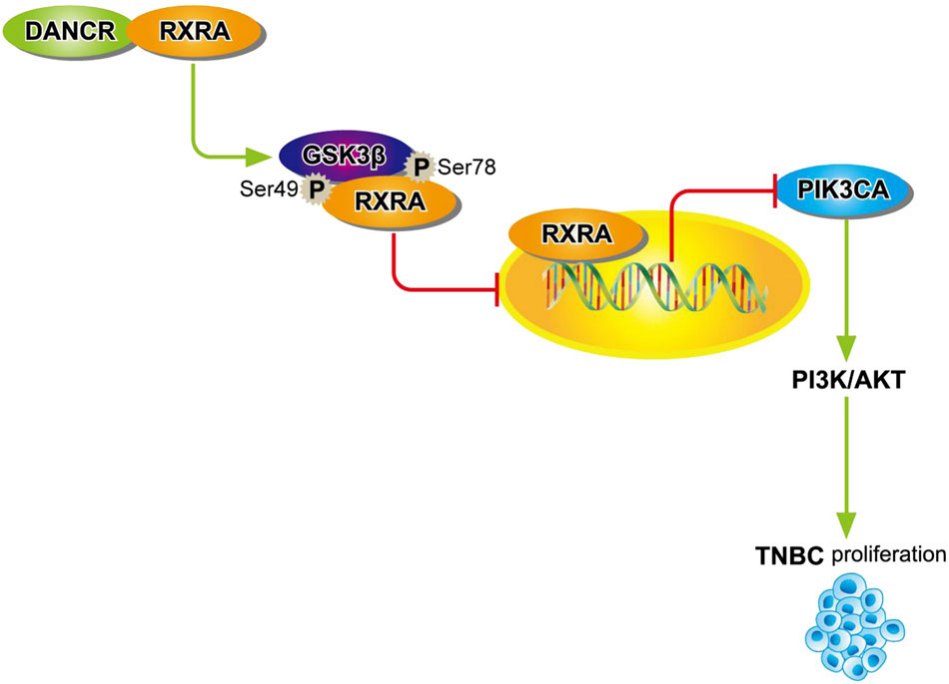
A working model for *DANCR*-mediated TNBC tumorigenesis. *DANCR* interacts with RXRA, and highly promotes GSK3β phosphorylating RXRA, and inhibits RXRA binding with PIK3CA promoter to upregulate its transcription, resulting in activating PI3K/AKT signaling pathway and TNBC tumorigenesis

In this study, we demonstrated a new mechanism by which *DANCR-*upregulated PI3K/AKT signaling pathway through activating serine phosphorylation of RXRA protein is important for TNBC cell proliferation and tumor growth (Fig. 8). By means of facilitating connection between RXRA and GSK3β, *DANCR* promotes the phosphorylation of RXRA, leading to suppress RXRA induced inhibition of PIK3CA transcription, thereby activating PI3K/AKT downstream signaling and ultimately promoting TNBC tumorigenesis.

Our data demonstrate that *DANCR* promotes TNBC tumorigenesis. *DANCR* was previously identified to play an important role in maintaining the undifferentiated cell state^15,16^. Previous work demonstrated that *DANCR* interacts with EZH2 to form a complex to promote stem cell characteristics^16,28^. It has been well documented that *DANCR* was overexpressed and amplified in breast cancer, and knockdown of *DANCR* significantly inhibited cell proliferation and invasion in breast cancer through facilitating binding of EZH2 to the promoters of ABCG2 and CD44 genes^14^. Here, we report that *DANCR* is upregulated in clinical TNBC samples, and higher *DANCR* level is positively correlated with poorer prognosis of TNBC patients. Knockdown of *DANCR* significantly inhibited TNBC cell proliferation, colony formation in vitro, and tumor growth in vivo. These results strongly support that *DANCR* is critical for TNBC tumorigenesis.

Our results also demonstrate that *DANCR* mediates TNBC through RXRA. RXRA had been reported to play critical roles in breast cancer cell progression^29,30^. Phosphorylation of the RXRA at serine 260 decreases its coactivator recruitment ability, leading to inhibition of RXRA transcriptional activity and enhanced cancer cell proliferation^31,32^. *DANCR* has also been identified as a molecular sponge mediating miR-758-3p^17^ and miR-577^18^ in non-small cell lung cancer and colorectal cancer, respectively. Furthermore, some studies showed that *DANCR* regulate breast cancer through binding and phosphorylating EZH2^25^. In this study, our data showed that *DANCR* directly bound with RXRA and mediated its phosphorylation. Knockdown of *DANCR* enhanced RXRA phosphorylation. Re-expression of shRNA resistant *DANCR* wild type impaired *DANCR* knockdown-promoted RXRA phosphorylation, whereas re-expression of shRNA resistant mutant type of the *DANCR*-RXRA binding did not affect it. Knockdown of RXRA rescued *DANCR* depletion-inhibited cell proliferation and soft agar colony formation. Taken together, our data demonstrate that *DANCR* regulate TNBC cell proliferation through RXRA phosphorylation.

Our results further suggest that *DANCR* mediates TNBC through RXRA- downregulating PI3K/AKT signaling pathway. Activating PI3K/AKT signaling was reported to be associated with poor OS in patients with breast cancer^33^. PIK3CA activation was also demonstrated to be critical for enhancing PI3K/AKT signaling^22^. RXRA was reported to mediate PI3K/AKT signaling in response to stem cell differentiation and provoke tumor suppression^34^. Inhibition of the N-terminally truncated RXRA association with the PI3K p85α subunit also resulted in suppression of PI3K/AKT signaling activation^35^. Here, we found that RXRA binds with the promoter of PIK3CA and downregulate PIK3CA transcription. We also observed that knockdown of *DANCR* inhibited RXRA protein phosphorylation and PIK3CA expression level. Interestingly, knockdown of RXRA rescued *DANCR* depletion-inhibited PIK3CA expression, cell proliferation, soft agar colony formation, and the promoter transcriptional activity of PIK3CA. Furthermore, We found *DANCR* markedly promotes RXRA association with GSK3β. In consistent with the previous study^31,32^, our data demonstrate that *DANCR-*induced RXRA phosphorylation suppresses RXRA-inhibited PIK3CA transcription in TNBC cells and ultimately activates the downstream PI3K/AKT signaling, whereas overexpression of GSK3β mutant type with deficient kinase activity or RXRA with mutated binding site with GSK3β dramatically blocked it. These data suggest that *DANCR* promoted TNBC tumorigenesis depends on its binding and phosphorylating RXRA, which leads to PI3K/AKT signaling pathway activation.

In summary, our results identified *DANCR* as an oncogene promotes TNBC tumorigenesis through a distinctive mechanism by which DANCR facilitated RXRA phosphorylation depending on GSK3β, thereby inhibiting the function of RXRA as a transcription repressors, and ultimately enhanced downstream PI3K/AKT signaling. Our findings have shed light into a novel roles of *DANCR* in TNBC tumorigenesis which have significant implications on better understanding the function of *DANCR* in human cancers. We hope that the newly established roles of *DANCR* and RXRA in tumorigenesis may provide a strong rationale for targeting them to improve the treatment of TNBC patients.

## Electronic supplementary material

Supplementry Figure 1

Supplementry Figure 2

Supplementry Figure 3

Supplementary Table 1

Rebuttal letter
