# Supplementary material for: LncRNA DANCR upregulates PI3K/AKT signaling through activating serine phosphorylation of RXRA
Source: Cell Death Dis. 2018 Dec 5;9(12):1167. doi: 10.1038/s41419-018-1220-7 (PMC6281578; doi:10.1038/s41419-018-1220-7)
Supplement: Supplementary file 1 — Supplementry Figure 1 [file 41419_2018_1220_MOESM1_ESM.pdf]

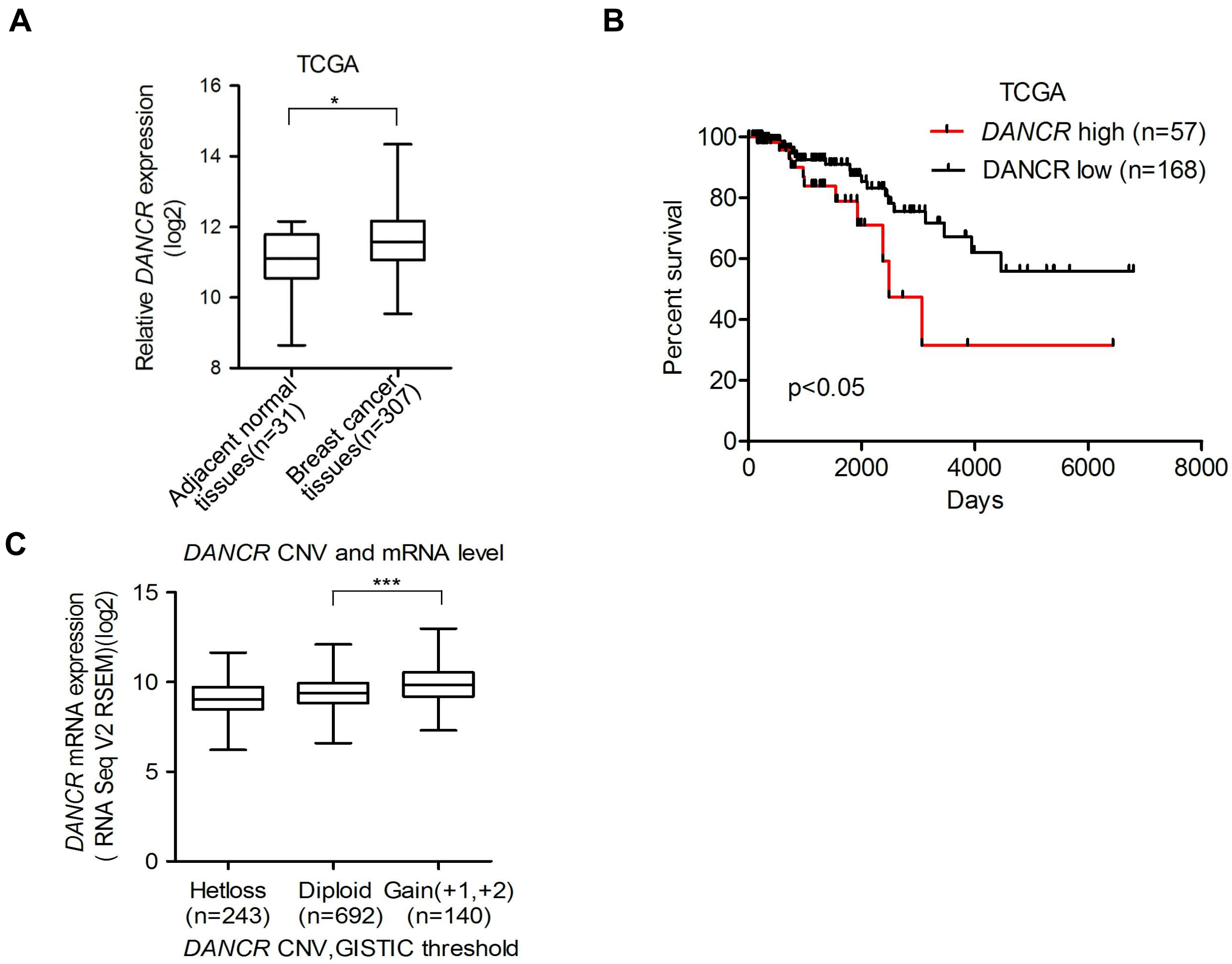

Supplementary Figure 1 Expression of *DANCR* in clinical breast cancer specimens

(A) *DANCR* mRNA expression levels are markedly higher in clinical breast cancer samples as compared to adjacent normal tissues. Data of *DANCR* mRNA expression were downloaded from the Cancer Genome Atlas (TCGA) microarray dataset. (B) Kaplan–Meier analysis of patients with high *DANCR* mRNA-expressing breast cancer versus low *DANCR* mRNA-expressing breast cancer from the TCGA microarray dataset. Statistical analysis was performed by log-rank test in a GraphPad Prism version 5.0 for Windows. (C) *DANCR* mRNA expression levels were markedly higher in samples with *DANCR* gained CNA compared with the samples without CNV in the TCGA dataset. Error bars ± SD. *, $P < 0.05$. *** $P<0.001$. Data are representative from two independent experiments.
