# Supplementary material for: LncRNA DANCR upregulates PI3K/AKT signaling through activating serine phosphorylation of RXRA
Source: Cell Death Dis. 2018 Dec 5;9(12):1167. doi: 10.1038/s41419-018-1220-7 (PMC6281578; doi:10.1038/s41419-018-1220-7)
Supplement: Supplementary file 2 — Supplementry Figure 2 [file 41419_2018_1220_MOESM2_ESM.pdf]

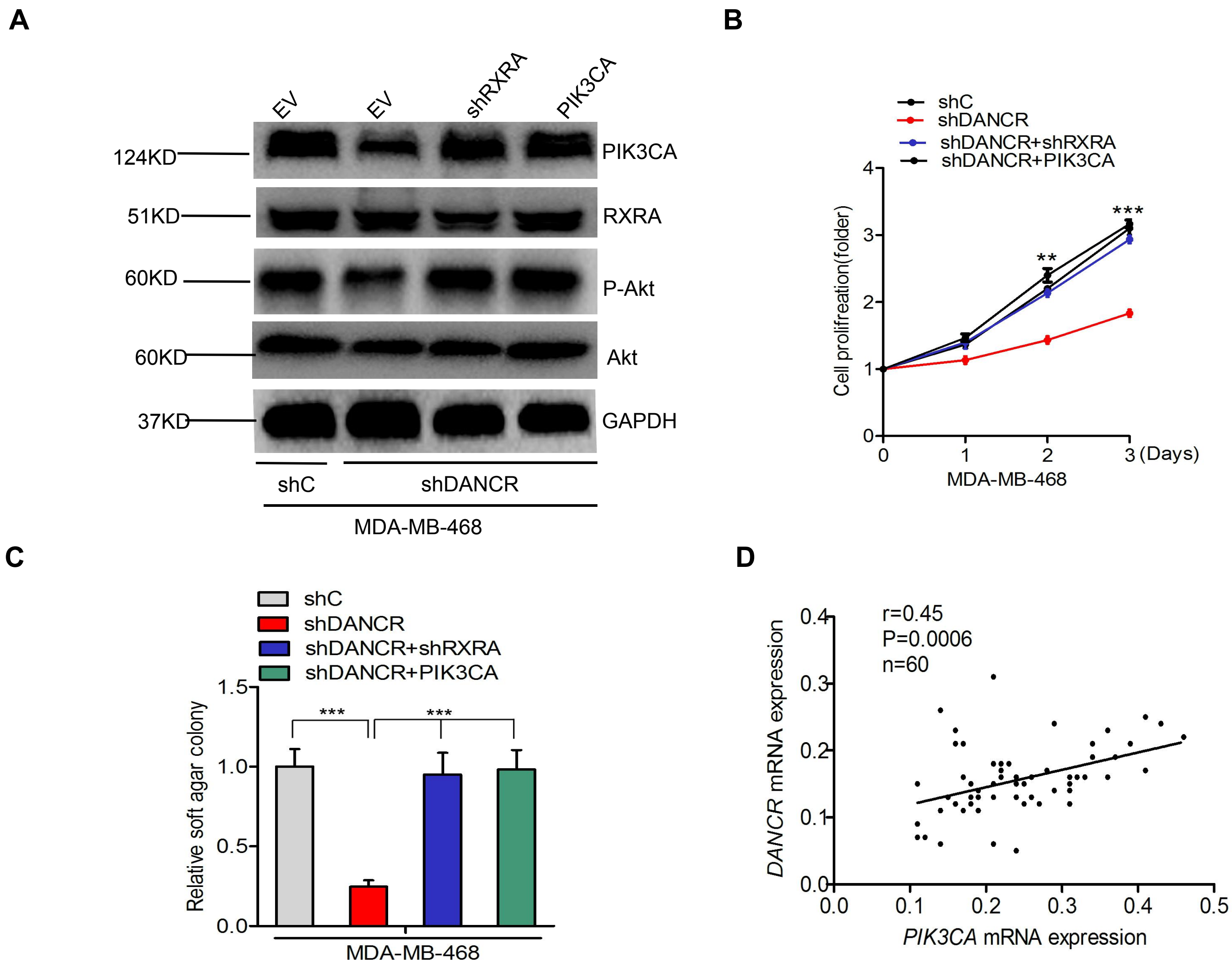

Supplementary Figure 2 *DANCR* facilitates PIK3CA transcription in a RXRA-mediated manner
(A) Knockdown of RXRA and overexpression of PIK3CA in *DANCR* depletion cells in MDA-MB-468 cells. (B and C) Knockdown of RXRA and overexpression of PIK3CA rescue *DANCR* knockdown-inhibited cell proliferation (B) and soft agar colony formation (C) in MDA-MB-468 cells. (D) Correlation analysis of between *DANCR* mRNA and PIK3CA mRNA in 60 TNBC samples.Error bars ± SD. **, P < 0.01. *** P<0.001. Data are representative from two independent experiments.
