# Supplementary material for: LncRNA DANCR upregulates PI3K/AKT signaling through activating serine phosphorylation of RXRA
Source: Cell Death Dis. 2018 Dec 5;9(12):1167. doi: 10.1038/s41419-018-1220-7 (PMC6281578; doi:10.1038/s41419-018-1220-7)
Supplement: Supplementary file 3 — Supplementry Figure 3 [file 41419_2018_1220_MOESM3_ESM.pdf]

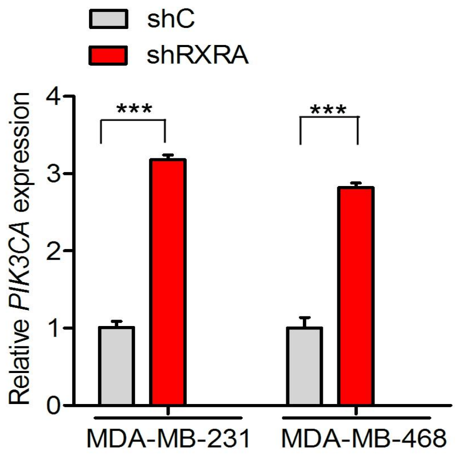

Supplementary Figure 3 RXRA regulates PIK3CA transcription

(A) Knockdown of RXRA increased PIK3CA mRNA in MDA-MB-231 and MDA-MB-468 cells. Error bars ± SD. *** $P<0.001$. Data are representative from two independent experiments.
