# Supplementary material for: LncRNA DANCR upregulates PI3K/AKT signaling through activating serine phosphorylation of RXRA
Source: Cell Death Dis. 2018 Dec 5;9(12):1167. doi: 10.1038/s41419-018-1220-7 (PMC6281578; doi:10.1038/s41419-018-1220-7)
Supplement: Supplementary file 4 — Supplementary Table 1 [file 41419_2018_1220_MOESM4_ESM.docx]

**Supplementary Table 1** Primers for qRT-PCR assays and ChIP-qPCR assays

| Primer pairs | Sequence |
| --- | --- |
| qRT-PCR,GAPDH | 5’-GGAGCGAGATCCCTCCAAAAT-3’ and  5’-GGCTGTTGTCATACTTCTCATGG-3’ |
| qRT-PCR,*DANCR* | 5’-TTGTATGGGTGCATGTTCAGC-3’ and  5’-TAAGTCAATTGAAATACCAGC-3’ |
| qRT-PCR,RXRA | 5’-ATGGACACCAAACATTTCCTGC-3’ and  5’-GGGAGCTGATGACCGAGAAAG-3’ |
| qRT-PCR,PIK3CA | 5’-CCACGACCATCATCAGGTGAA-3’ and  5’-CCTCACGGAGGCATTCTAAAGT-3’ |
| DANCR shRNA #1 | 5’-TGGAGCTAGAGCAGTGACAATGCGAACATTGTCACTGCTCTAGCTCCTTTTC-3’and  5’-TCGAGAAAAGGAGCTAGAGCAGTGACAATGTTCGCATTGTCACTGCTCTAGCTCCA-3’ |
| DANCR shRNA #2 | 5’-TGGTCACCAGACTTGCTACACCCGAAGGTGTAGCAAGTCTGGTGACCTTTTC-3’ and  5’-TCGAGAAAAGGTCACCAGACTTGCTACACCTTCGGGTGTAGCAAGTCTGGTGACCA-3’ |
| RXRA shRNA #1 | 5’-TGGCAAGCACTATGGAGTGTACCGAAGTACACTCCATAGTGCTTGCCTTTTC-3’and  5’-TCGAGAAAAGGCAAGCACTATGGAGTGTACTTCGGTACACTCCATAGTGCTTGCCA-3’ |
| ChIP-qPCR,PIK3CA  -1937 to -1691 | 5’-ATGTTGGCTGGTGCCTG-3’ and  5’-AGCACAGAGTTATATGAG-3’ |
| ChIP-qPCR,PIK3CA  -1604 to -1341 | 5’-AATCTGTACTCTGGAG-3’ and  5’-TACTTGTCCTCTAGAGC-3’ |
| ChIP-qPCR,PIK3CA  -1201 to -967 | 5’-ATTACAACAAAAGACC-3’ and  5’-CTGATTCGAAGCAGCT-3’ |
| ChIP-qPCR,PIK3CA  -908 to -641 | 5’-AGGACTGCAGAGGGC-3’ and  5’-GCGAGCTGGGGTAGA-3’ |
| ChIP-qPCR,PIK3CA  -587 to -301 | 5’-CACCCTCACTACTGCAG-3’ and  5’-CACATCGTGTAAACAA-3’ |
| ChIP-qPCR,PIK3CA  -286 to -12 | 5’-CCAATAAAGTTTATTC-3’ and  5’-GGAGAGCGGGCGGCAC-3’ |
