# Supplementary material for: LncRNA DANCR upregulates PI3K/AKT signaling through activating serine phosphorylation of RXRA
Source: Cell Death Dis. 2018 Dec 5;9(12):1167. doi: 10.1038/s41419-018-1220-7 (PMC6281578; doi:10.1038/s41419-018-1220-7)
Supplement: Supplementary file 5 — Rebuttal letter [file 41419_2018_1220_MOESM5_ESM.docx]

**Rebuttal letter**

“LncRNA DANCR upregulates PI3K/AKT signaling through activating serine phosphorylation of RXRA”- CDDIS-18-3001-T

Dear editor

Thank you for your letter and for the Referees’ comments concerning our manuscript “LncRNA DANCR upregulates PI3K/AKT signaling through activating serine phosphorylation of RXRA”. The comments are all valuable and very helpful for revising and improving our paper, as well as the important guiding significance to researches. We studied the comments carefully and have made corrections which we hope meet with approval. The article has been re-edited and some figures and figure legends have been modified to have a better understanding. And the red font was marked to indicate the revised portions in our manuscript. Some supplements of the new experimental data that the Referee suggested have been added in the manuscript.

Please let me know if you have any questions or need further information. Thank you for your assistance.

Sincerely

Aihong Bi

Department of Radiation Oncology, Zhejiang Provincial People's Hospital

People's Hospital of Hangzhou Medical College

***************************************************************

**The reply to the suggestions from Referees:**

Reviewer #1 (Comments to the Author):

1. An interaction between DNACR and RXRA in TNCB cells was shown. I was wondering if this is a direct interaction or whether it is mediated by another protein.

Thanks for your valuable concern. The previous researches have provided much related information. It is reported that lncRNA can directly interact with proteins thus affecting its function. For example, lncRNA EPIC1 can promotes cell-cycle progression by directly binding with MYC through EPIC1’s 129-283 nt region to regulate its downstream target gene expression (Wang Z et al. Cancer Cell, 2018). Another study also reported that lncRNA lnc-DC can bind directly to STAT3, a transcription factor, which promoted STAT3 phosphorylation (Wang P et al. Science, 2014). In our study, though RNA pull-down assay and RIP-qPCR, we clearly demonstrated that DANCR directly bound to RXRA. Furthermore, through deletion-mapping methods, we directly identified a 300nt region at the 5’ end of DANCR required for RXRA binding.

Importantly, the referee’s concern are valuable and very inspirational for our further study. We cannot rule out other proteins involved in this process, which are worthy of further investigation.

2. While overall the mechanistic link between DANCR activity and RXRA phosphorylation and the downstream consequences are convincing, I felt that in some of the presented WB (for example- Figures 4C, 4D, 5C, 7B and 7C) lower exposures will be more informative. It would be helpful if the authors can provide it. Alternatively, quantification of the bands intensity (normalize to GAPDH) will also be sufficient here.

Thanks for your valuable advice. As suggested by the referee, we replaced the WB images (Figures 4C, 4D, 5C, 7B and 7C) with lower exposures, and the quantification of GAPDH bands intensity was also added.

3. The authors nicely show by ChIP and luciferase assays that RXRA binds to the PIK3CA promoter and represses PIK3CA promoter. Measuring the endogenous level of PIK3CA transcript by regular qPCR will make their claim stronger.

The comments are valuable and very helpful for our study. The experiments and results were added in Supplementary Figure 3.

4. Fig 1D- Y axis which represent the percent survival should be up to 100 and not 150.

Thank you for the suggestion, we corrected this error and apologize for the inconvenience caused.
